# Impact of Heatwaves on the Physiology and Retail Meat Quality of Lambs

**DOI:** 10.3390/foods11030414

**Published:** 2022-01-31

**Authors:** Minghao Zhang, Robyn D. Warner, Frank R. Dunshea, Kristy DiGiacomo, Aleena Joy, Archana Abhijith, Pragna Prathap, Ting Ma, Surinder S. Chauhan

**Affiliations:** 1 School of Agriculture and Food, The University of Melbourne, Melbourne 3010, Australia; minghao@student.unimelb.edu.au (M.Z.); robyn.warner@unimelb.edu.au (R.D.W.); fdunshea@unimelb.edu.au (F.R.D.); kristyd@unimelb.edu.au (K.D.); aleenajoyj@student.unimelb.edu.au (A.J.); apayyanakkal@student.unimeb.edu.au (A.A.); pprathap@student.unimelb.edu.au (P.P.); tingm1@student.unimelb.edu.au (T.M.); 2 Faculty of Biological Sciences, The University of Leeds, Leeds LS2 9JT, UK

**Keywords:** heat stress, lamb meat, retail display, physiological performance

## Abstract

The experiment investigated the impact of heatwaves (HWs) on the physiology, postmortem muscle metabolism and meat quality of lambs. Seventy-two second-cross lambs (Poll Dorset × (Border Leicester × Merino)) were selected and exposed to either 1, 3 or 5 days of HWs or thermoneutal (TN) (28–38 °C and 40–60% relative humidity, RH; 18–21 °C, 40–55% RH) conditions in climate-controlled chambers. Lambs exposed to 1–5 days of a HW exhibited higher respiration rates (RRs), rectal temperatures (RTs), skin temperatures (STs) and heart rates (HRs) compared to lambs exposed to an equal duration of TN conditions. However, HWs had no significant effects on muscle metabolism (rate and extent of pH decline, muscle glycogen and lactate content) and meat quality (cooking loss and shear force). Similarly, there were limited impacts of 1–5 days of HW on the colour (L*, a*, b* and R630/580) and drip loss of the longissimus thoracis et lumborum (LTL) and semimembranosus (SM) muscles for 4 days’ overwrap retail display. Results suggest that short-duration HWs (1–5 days) had significant negative effects on animal physiology but had no effect on the muscle metabolism and meat quality.

## 1. Introduction

Heat stress (HS) is one of the biggest challenges facing animal production over the summer months and is likely to be accentuated by climate change and global warming. High ambient temperature and humidity compromise animal production and welfare as animals cannot maintain core body temperature due to the extra heat load [1]. Research over the last decade has suggested that meat quality problems, such as dark-cutting (high ultimate pH) or pale soft exudative (PSE, rapid pH fall post-mortem) meat, are more prevalent in summer [2,3,4]. However, direct impacts of HS on meat quality are not well understood and may vary depending upon the duration and severity of ambient temperature and animal species and breed [3]. A higher incidence of dark-cutting meat has been reported in small ruminants such as sheep and goats when the heat exposure period is longer than 1 month, resulting in lower feed intake and decreased body weight gain [2,5,6]. However, the effects of short-duration heat events, such as those experienced during a heatwave (HW), on sheep meat quality remain unknown.

Previously, Lowe et al. [7] reported that 12 h HS (33 °C, 85–100% RH) did not change the meat quality of Romney cross ewe lambs. Our study recently showed that 1 and 2 weeks of cyclic heat stress (28–40 °C, 40–60% RH) did not impact lamb meat quality [8,9]. However, recent climate data shows that 1–5 days are the most likely duration of summer HW in the majority of countries, including Australia [10], China [11] and the U.S. [12]. Furthermore, there are growing concerns that global warming will further aggravate the challenge of HS as the HWs are getting hotter, longer and more frequent [10]. Therefore, understanding the impacts of short-term heat events such as HWs on meat quality are required. This study was designed to investigate the potential effects of a simulated HW experiment on small ruminants’ meat quality. The specific objective of this experiment was to assess the impact of three different HW durations (1 day, 3 days and 5 days) on the physiological and metabolic biomarkers, postmortem muscle metabolism and meat quality of lambs. We hypothesized that HWs will have negative impacts on the physiological parameters, which include RR, RT, HR and ST, and on the meat quality (glycolysis, colour, WHC and texture) of lambs, irrespective of the duration of the HW. The experiment result would further help estimate the economic losses associated with HWs and guide policy and decision making to develop appropriate strategies to reduce the negative impacts of HS and improve animal welfare and product quality.

## 2. Materials and Methods

This experiment and all treatment procedures for live animals were assessed and approved by the University of Melbourne Faculty of Veterinary and Animal Sciences Animal Ethics Committee (AEC ID 1914955.1).

### 2.1. Animals and Experimental Design

Seventy-two second-cross female lambs (Poll Dorset X (Merino X Border Leicester)) aged between 9–12 months and weighing 49.0 ± 7.0 kg were procured from 3 different breeders in Northeast Victoria. The experiment was conducted using a randomized 2 × 3 factorial design with 6 consecutive replications. Lambs in each replication were randomly allocated to either HW or thermoneutral (TN) conditions for 1, 3, and 5 days (12 lambs for each group, 3 groups for HW and 3 groups for TN). Lambs were acclimatized to indoor group feeding (2 weeks) followed by 1 week of individual pen feeding before relocation into metabolism cages (1.0 × 0.5 m with polypropylene slat flooring that has a stable grip preventing sheep from slipping) and exposed to simulated HWs or TN conditions in the climatic chambers. 

**Figure 1:**
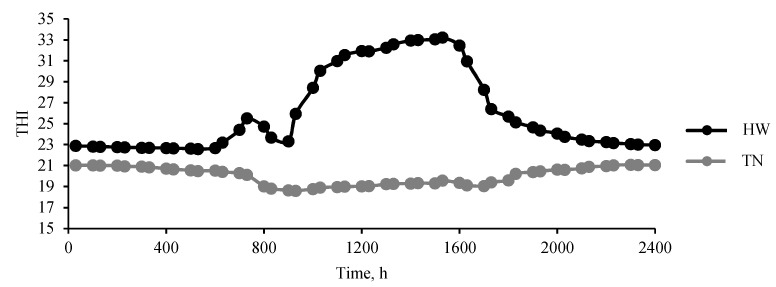
Average daily temperature humidity index (THI) recorded in the heatwave (HW) and the thermoneutral room (TN) during the experimental period. The average THI of the HW was 30.1 in the daytime and 23.9 in the nighttime (standard error difference of means = 0.55) and the THI of the TN was 19.8 (standard error difference of means = 0.67). THI < 22.2 = no heat stress, 22.2 to 23.3 = moderate heat stress, 23.3 to 25.6 = serve heat stress, >25.6 = extreme severe heat stress [3,13].

After 3 days of acclimatization in the climatic chambers, lambs were exposed to either TN (18–21 °C, 45–55% RH, *n* = 12) or to a HW (28 °C (16:00 to 8:00 h) and 38 °C (8:00 to 16:00 h), 40–60% RH, *n* = 12) for different thermal treatment times (1, 3 and 5 days) according to the experimental design. In the HW room, the heaters and humidifiers were turned on at 8:00 h to maintain the room temperature between 36 and 38 °C and RH between 40% and 60% until 16:00 h. After 16:00 h, the heater was turned off, and the air conditioning system set to maintain the room temperature between 26 and 28 °C overnight until the next day at 8:00 h. Room temperature and RH were recorded every 30 min by temperature-humidity data loggers (TechBrands, Electus Distribution, Rydalmere, Australia; mounted at 1 m height), and the temperature-humidity index (THI) was calculated using the following equation: THI = db °C − (0.31 − 0.31 RH) × (db °C − 14.4) [13] and is presented in Figure 1 for the two treatments. Lambs were individually fed, and water was available throughout the day. The diet consisted of 25% oaten chaff and 25% lucerne chaff, combined with standard lamb finisher pellets which was formulated according to NRC, 2007 (50% pellets: 14% protein, 8% crude fibre, 2% added salt, 1% added urea). Lambs were fed ad libitum with 2 × maintenance requirements per day. The equation: feed intake (kg DM/day) = W^0.75^ × 450/1000/ME (maintenance energy) was used to calculate the animals’ daily feed requirements throughout the experiment.

### 2.2. Physiological Parameters

Respiration rate (RR) was measured by counting the flank movements in 20 s, and heart rate (HR) was measured with a stethoscope for 20 s, then converted to RR or HR per minute. Skin temperature (ST) and Rectal temperature (RT) at the right flank were measured with a digital thermometer (EN60601, Welcare, SA, Australia). For skin temperature, the thermometer was placed in contact with the skin until the temperature reading stabilized. Both physiological parameters (HR, RR, RT and ST) were recorded at 8:00 h, 12:00 h and 16:00 h daily throughout the treatment period.

### 2.3. Slaughter

At the end of each treatment period (day 1, 3 or 5 according to the treatment group), lambs were slaughtered using a licensed mobile abattoir after 12 h fasting, with access to water. The slaughter procedure was followed as standard commercial operations, including captive bolt stunning. The carcass was chilled in a mobile chiller at 0 to 4 °C for 24 h after slaughter, then the longissimus thoracis et lumborum (LTL) and semimembranosus (SM) muscles were removed from both sides of the carcass. Muscles were vacuum-packed, then stored at 0–4 °C in the mobile chiller until further measurements.

#### Postmortem Muscle Sample Collection

After slaughter and exsanguination, 30 g-sample were collected from the Longissimus lumborum (LL) muscle at 5 min, 1 h, 2 h, 3 h, 4 h, 5 h, 12 h and 24 h postmortem. Muscle samples were snap-frozen in liquid nitrogen and stored in the freezer (−80 °C) for pH and metabolite analysis.

### 2.4. Meat Quality

#### 2.4.1. Meat Packaging and Retail Display

At 48 h postmortem, LTL and SM muscles were cut into 5 pieces (140 g) and packaged for retail display. Samples were vacuum-packed for 4 days’ aging and placed on a cello pad positioned in Cryovac black trays after aging (170 mm × 223 mm, Sealed Air, Sydney, NSW, Australia). Trays were packaged with oxygen-permeable polyvinyl chloride film (Food cling wrap, Glad, Oakland, CA, USA). Meat samples were subsequently kept in a 4–6 °C in a refrigerator (display cabinet) with high-impact LED internal lighting on each side (maximum 18 W: GM1000LWCAS, Bromic, Sydney, NSW, Australia) for 4 days’ retail display. Meat colour was measured every 24 h for 4 days’ display, and water holding capacity (WHC) and Warner–Bratzler shear force (WBSF) were measured before and after 4 days’ display.

#### 2.4.2. Surface Colour

Meat colour (CIE-1986 mode [14], lightness (L*), redness/greenness (a*), and yellowness/blueness (b*)) of muscle surface was measured in triplicate using a Hunter lab Miniscan™ XE Plus 45/10 colorimeter (Reston, VA, USA). Measurements were made on day 0, day 1, day 2, day 3 and day 4 on removal from the retail display case. The light source was set at illuminant D65 with the 10° standard observer R-300 with 8 mm aperture, and the average of three readings were recorded [15]. Ratios between the reflectance at 630 nm and 580 nm (R630/580) were used to estimate the oxy/met ratio (also called redness in some papers) [16]. This ratio is a measure of meat colour stability over time. 

#### 2.4.3. Water Holding Capacity

Water holding capacity (WHC) was determined by measuring drip loss, cooking loss and purge loss of LTL and SM muscle samples at 0- and 4-days’ display. Muscle drip loss was measured before retail display using the EZ-drip loss method and tubes purchased from Danish meat (DK) [9,17]. Muscle samples of 10 g and 17 cm thickness were collected by a circular knife. Then the sample weight was weighed as W1 and kept in the EZ-drip loss container at 4–6 °C. After 48 h, samples were weighed again (W2), and the drip loss was calculated as: Drip loss (%) = {(W1 − W2)/W1} × 100.

After colour measurements, meat samples were used for purge and cooking loss measurements. For purge loss, 90 g of muscle sample was weighed before (W1) and after (W2) 4 days’ display. After purge loss weighing, the same muscle samples were cooked in plastic bags in a temperature-equilibrated water bath (75 °C; F38-ME, Julabo, Seelbach, Germany) until the core temperature reached 71 °C. The core temperature of muscle samples was monitored with a Grant thermometer equipped with T-type thermocouples (Grant Instruments, Cambridge, UK) during cooking. After cooking, muscles were cooled in iced water, stored at 0–4 °C in the mobile chiller for 12 h, then weighed and recorded as W3 [18]. Purge and cooking loss calculation was calculated using the equation as follows: Purge loss (%) = {(W1 − W2)/W1} × 100/Cooking loss (%) = {(W2 − W3)/W2} × 100.

#### 2.4.4. Warner–Bratzler Shear Force

After cooking-loss weighing, cooked samples were subjected to Warner–Bratzler peak shear force using the Lloud texture analyzer (TA-1, Lloyd Instruments, AMETEK, Berwyn, IL, USA), with the measurement protocol and setting adapted from previous established protocols by Minh Ha et al. [19]. Each sample was cut into 6 sub-samples (1 cm × 1 cm × 4 cm) with the direction of muscle fibers running longitudinally. The shear blade (V-shaped) was used for WBSF measurements with a 500 N load cell (300 mm/min shearing speed). Each sample was measured with 6 tests, and the average of 6 measurements was presented. 

### 2.5. Muscle pH, Glycogen and Lactates

For determining the pH decline and glycolytic metabolite concentration in the muscle samples, muscle samples were collected at 5 min, 1 h, 2 h, 3 h, 4 h, 5 h, 12 h and 24 h postmortem, snap-frozen, then ground using a tissue grinder (20 s, 30 times/s). The pH of the muscle sample was measured using previously reported methods [20] with a slight modification [21]. Briefly, 100 mg of powdered muscle sample was homogenized with 800 μL 5 mM iodoacetic acid and 150 mM KCl (pH = 7.0) buffer. After homogenization, the sample was centrifuged at 10,000× *g* for 5 min. The pH was measured using a combined pH and temperature meter (WP-80M, TPS, Brendale, Australia) equipped with a spear-head pH probe (IJ44C probe, TPS, Brendale, Australia) and calibrated using 7.0 and 4.0 pH buffers. For glycogen and lactate, 100 mg muscle samples were homogenized with 1.0 mL distilled water, and then the sample was centrifuged at 13,000× *g* for 5 min (glycogen) or 10 min (lactate) to remove insoluble material [22]. The supernatant was used for glycogen and lactate concentration measurements using commercial assay kits (MAK016/MAK064, Sigma, San Jose, CA, USA). 

### 2.6. Statistical Analysis

Statistical analysis adapted linear mixed model (REML) procedures in the GenStat 18th edition. For physiological parameters (RT, RR, HR, ST), fixed model effects were temperature (HW and TN), duration of temperature treatment (1, 3 or 5 days), and time of the day (8:00, 12:00, 16:00 h). For the analysis of glycolysis parameters (muscle pH, glycogen and lactate), fixed factors were temperature (HW and TN), treatment (1, 3 or 5 days) and postmortem time (only for pH decline). For meat quality parameters, temperature, treatment days and retail display times (0, 1, 2, 3 and 4 days) were the fixed factors. Replication and sheep ID were used as random terms in the model for all analysis. Results are reported as means and standard errors of difference. Means were considered to differ significantly when *p* ≤ 0.05, and group comparisons were conducted using LSD values. 

## 3. Results and Discussion

### 3.1. Temperature–Humidity Index and Physiological Parameters

Temperature–humidity index (THI), which is calculated based on the ambient temperature and the relative humidity, is commonly used to measure heat stress. In this experiment, the average THI in the simulated HW room was 30.1 in the daytime and 23.9 at night, indicating that the lambs were exposed to high-temperature conditions during the day and were not able to offload heat overnight (Figure 1). As per the THI developed exclusively for sheep, a THI lower than 22.2 is classified as thermoneutral, a THI of 22.2 to 23.3 is moderate HS, a THI of 23.3 to 25.6 is classified as severe HS, and when THI exceeds 25.6 it is considered extremely severe HS [13,23]. 

**Figure 2:**
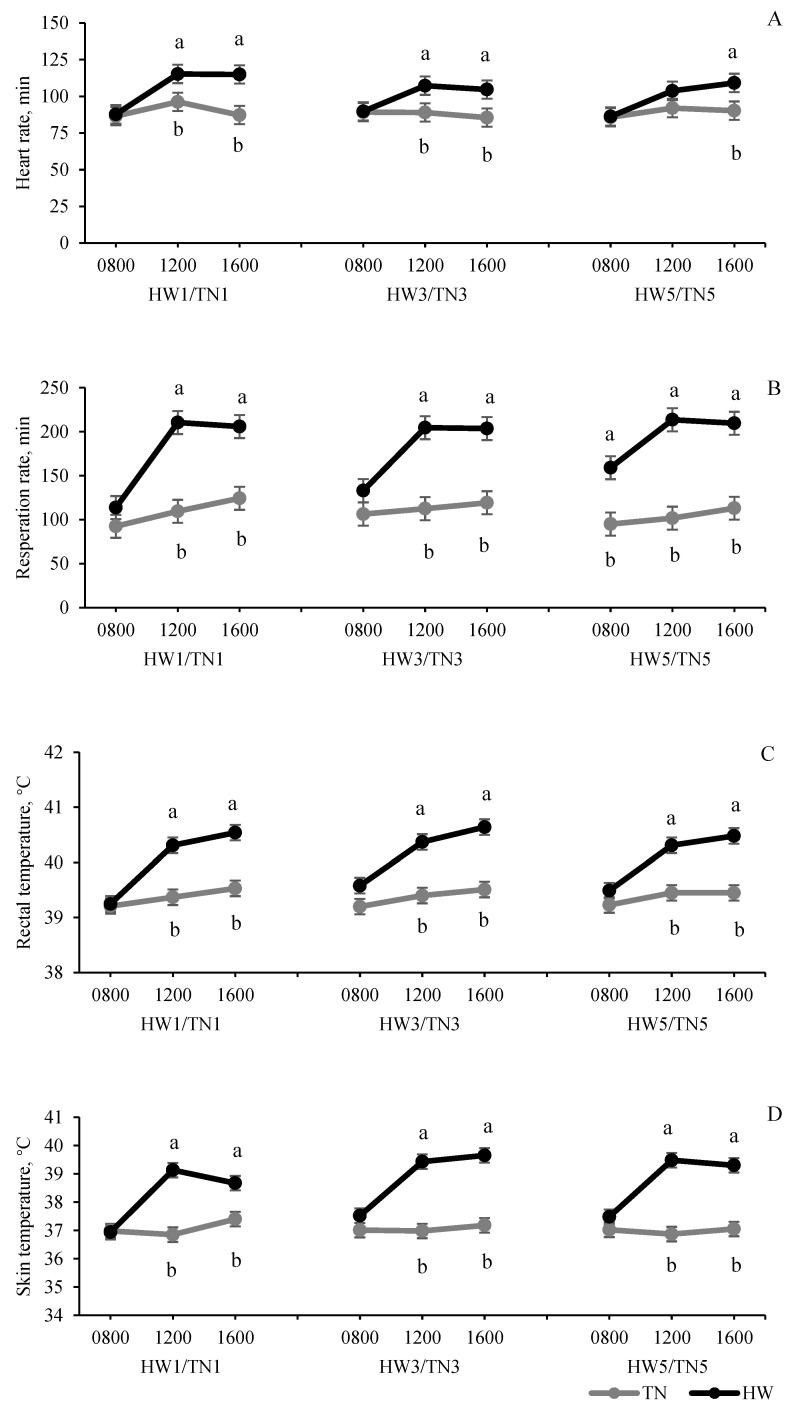
The effect temperature (thermoneutral, TN vs. heatwaves, HW) and heat exposure duration (1 vs. 3 vs. 5 days) have on physiological parameters of second-cross lambs at different times (8:00 h, 12:00 h, 16:00 h) on the last experiment day (Day 5); values are mean ± SED; a and b indicate levels that are significantly different at 5% level of LSD in the same timepoint; HW/TN1–5 = 1 to 5 days HW/TN groups (*n* = 12 per group); heart rate, temp. < 0.001; day *p* = 0.22; temp. × day *p* = 0.43 respiration rate, temp. *p* < 0.001; day *p* = 0.54; temp. × day *p* = 0.055; rectal temperature, temp. *p* < 0.001; day *p* = 0.65; temp. × day *p* = 0.65; skin temperature, temp. *p* < 0.001; day *p* = 0.080; temp. × day *p* = 0.062. (**A**) Heart rate; (**B**) Respiration rate; (**C**) Rectal temperature; (**D**) Skin temperature.

Respiration rate, rectal and skin temperature, and heart rate responses to HW exposure are presented in Figure 2. Overall, there was a significant (*p* < 0.01) effect of HWs on all lamb physiological parameters (RR, RT, ST and HR) such that lambs exposed to 1, 3, and 5 days of a HW exhibited higher RR, RT, ST and HR than their counterparts in the TN group at 12:00 h and 16:00 h. Lambs exposed to 5 days of a HW showed a significant increase in their resting respiration (recorded at 08:00 h) as compared to the lambs in TN group (*p* < 0.05), but this increase was not observed in the 1 and 3 days’ group.

The increase in RR, RT and ST with exposure to a HW observed in this experiment agrees with previous research on HS in sheep [9,24,25,26]. The increase in RT is a commonly used indicator of HS observed when ambient temperature exceeds the species temperature threshold, and the animal can no longer maintain its core body temperature. The increase of RT in all HW (1, 3 and 5) groups as compared to TN sheep showed that a HW, irrespective of duration, perturbs the normal physiology of sheep and compromises their ability to regulate core body temperature. RR is increased by exposure to hot conditions in an effort to increase heat loss from the body as heat dissipation via water vapor in exhaled air is increased up to 60% in HS conditions compared to 20% in TN conditions [25]. RR and RT are considered standard physiological parameters to measure HS response in animals. Generally, sheep are considered to be suffering HS when RR reaches 200 breaths/min [27,28] and body temperature exceeds 39.9 °C [29]. In this experiment, all HW groups (1 to 5 days) reached 200 breaths/min at 12:00 h and 16:00 h. As the heat load starts to accumulate in the animals with the increased duration of HW exposure, the basal RR also gradually increases as was observed in this study with the RR in the morning (08:00 h) increasing from 1 day to 5 days’ HW exposure. The difference in RR between the 5-days HW and TN groups at 08:00 h (*p* < 0.05) showed that lambs accumulated more heat with heat exposure for 5 days as compared with 1 and 3 days’ HW exposure, which typically happens with a HW because the lamb could not offload the heat-load during the warm night. 

HWs also increased lambs’ heart rates. The heart rate of the 1- and 3-day HW groups were significantly higher than the corresponding TN groups at 12:00 h and 16:00 h, and the 5-days HW group was higher than the TN group (5 days) at 16:00 h (Figure 2; *p* < 0.05). Heart rate is another important physiological parameter that is increased by exposure to high ambient temperature conditions to increase blood flow to the extremities to promote heat loss from the body. This increased blood flow helps sheep transfer heat from the interior of the body into the subcutaneous layer [30]. However, if the exposure to hot conditions is prolonged, HR may decrease due to peripheral vasodilation and lower metabolic rate under HS conditions [31]. 

### 3.2. Postmortem Muscle Glycolysis and Meat Quality

After animals are slaughtered for meat, muscle glycogen is utilized under anaerobic conditions to achieve premortem homeostatic balance. This glycolytic metabolism results in the consumption of glycogen and the formation of lactate and H+ ions, resulting in muscle pH decline from about 7.0 premortem to an ultimate pH of about 5.5. Any deviation from the normal rate and extent of postmortem muscle metabolism or glycolysis may lead to poor meat quality development, such as faster pH decline, resulting in PSE meat [28] or meat with a higher pHu, called dark cutting in ruminants [30].

**Figure 3:**
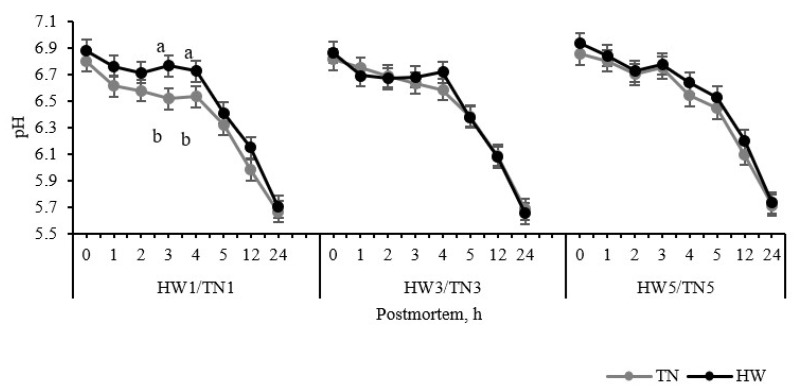
The effect of temperature (thermoneutral, TN vs. heatwaves, HW) and heat exposure duration (1 vs. 3 vs. 5 days) on pH drop of the longissimus thoracis et lumborum muscle at 5 min, 1 h, 2 h, 3 h, 4 h, 5 h, 12 h, 24 h postmortem: values are mean ± SED; a and b indicate levels that are significantly different at 5% level of LSD in the same timepoint; HW/TN1–5 = 1 to 5 days HW/TN groups (*n* = 12 per group); pH: temp. *p* = 0.12; day *p* = 0.20; temp. × day *p* = 0.31.

In this experiment, all HW treatments (1 to 5 days) had no impact on the initial pH of LTL muscle (*p* > 0.05) recorded 5 min postmortem (Figure 3). Regarding the pH decline over the 24 h postmortem period, only the 1-day HW group showed higher pH value (slower rate of pH decline) than the 1-day TN group at 3 h and 4 h postmortem (*p* < 0.05). In contrast, the ultimate pH of both groups was the same (*p* > 0.05). There was no significant difference (*p* > 0.05) between different heat exposure times, and the interaction between temperature and time was not significant. Similar results were observed for postmortem muscle lactate and glycogen concentration, and there were no effects of HW on lactate and glycogen concentration in the LTL muscle samples collected at 24 h postmortem (*p* > 0.05).

**Figure 4:**
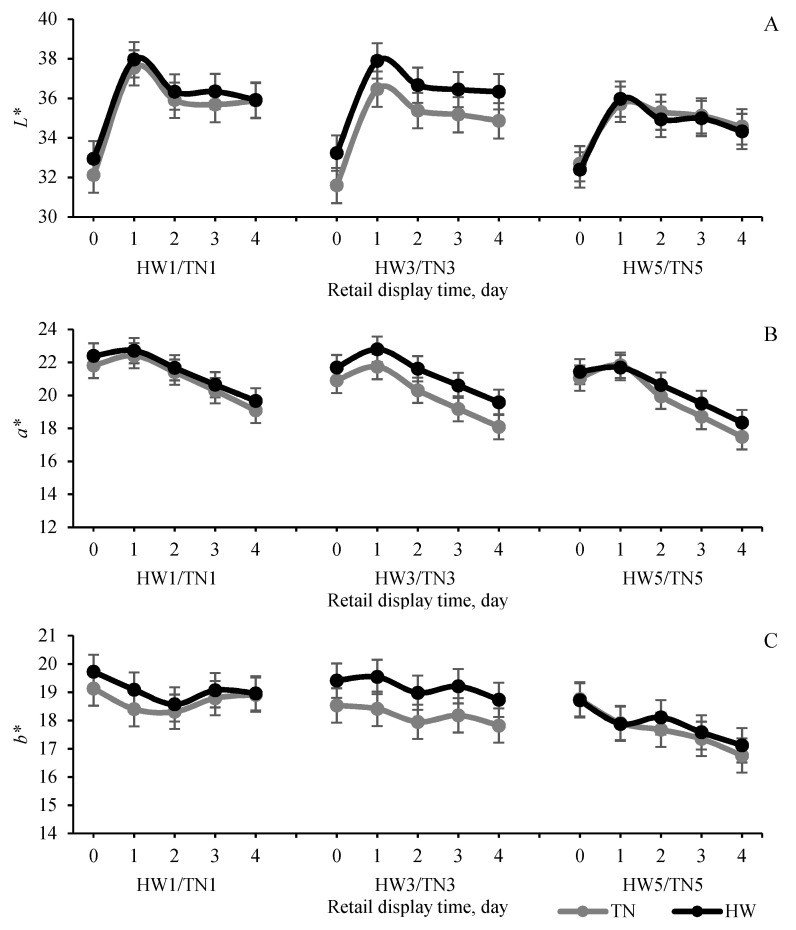
The effect of temperature (thermoneutral, TN vs. heatwaves, HW) and heat exposure duration (1 vs. 3 vs. 5 days) on lightness (L*), redness (a*), yellowness (b*) of longissimus thoracis et lumborum muscle in 4 days’ overwrap retail display: values are mean ± SED. HW/TN1–5 = 1 to 5 days HW/TN groups (*n* = 12 per group); lightness (L*; temp. *p* = 0.24; day *p* = 0.19; temp. × day *p* = 0.43); redness (a*; temp. *p* = 0.046; day *p* = 0.054; temp. × day *p* = 0.68); Yellowness (b*: temp. *p* = 0.046; day *p* = 0.001; temp. × day *p* = 0.68). (**A**) lightness (L*); (**B**) redness (a*); (**C**) yellowness (b*).

**Figure 5:**
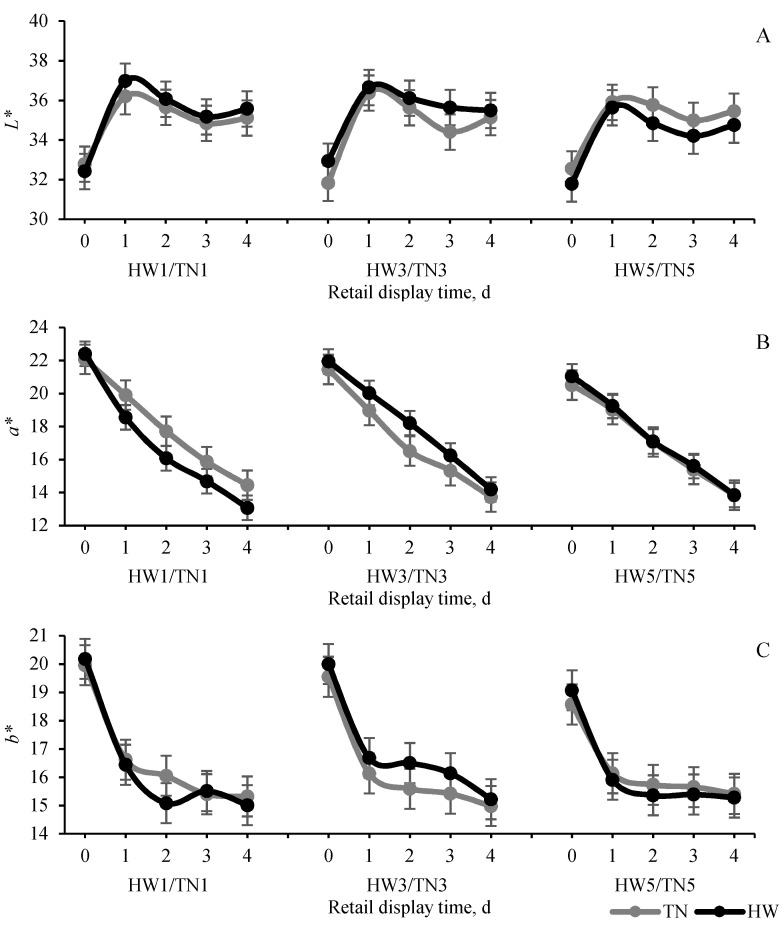
The effect temperature (thermoneutral, TN vs. heatwaves, HW) and heat exposure duration (1 vs. 3 vs. 5 days) on lightness (L*), redness (a*), yellowness (b*) of semimembranosus muscle in 4 days’ overwrap retail display: values are mean ± SED; HW/TN1–5 = 1 to 5 days HW/TN groups; temp. = temperature (n = 12 per group); lightness (L*; temp. *p* = 0.73; day *p* = 0.64; temp. × day *p* = 0.42); redness (a*; temp. *p* = 0.95; day *p* = 0.51; temp. × day *p* = 0.054); yellowness (b*: temp. *p* = 0.62; day *p* = 0.39; temp. × day *p* = 0.35). (**A**) lightness (L*); (**B**) redness (a*); (**C**) yellowness (b*).

Unlike the stress response observed in physiological parameters and similar to postmortem muscle glycolytic results, HWs had a very limited impact on retail meat colour performance and stability for both the LTL and SM muscles (Figure 4 and Figure 5). HWs had a significant effect only on a* and b* values of LTL muscle (*p* < 0.05). The 3-day HW group had higher a* and b* values than the 3-day TN group. There was a significant correlation between temperature and retail display days for a* (*p* = 0.054) of the SM such that the 1 day HW group showed faster discoloration (lower a* in 4 days’ display) than the 3- and 5-day HW groups. There was no HW effect on R630/580 of the LTL and SM muscles. Similar to meat colour, HWs had limited influence on WHC and shear force (Table 1 and Table 2) of both the LTL and the SM. All (1 to 5 days) HW groups had significant impact on drip loss of the LTL and drip loss and purge loss of the SM muscle (*p* < 0.05). For the LTL muscle, drip loss in 5 days in the TN group (1.95%) was significantly higher than in the HW group (1.33%), but the difference was not observed in the 3- and 1-day groups. Similar to the LTL muscle, the SM muscle drip loss and purge loss in the 1-day HW group was higher than in the 1-day TN group but 3- days groups had no difference. There was no difference in cooking loss between TN and HW groups before and after 4 days’ retail display (*p* > 0.05). Similarly, HW had no influence on shear force of the LTL and SM muscle except in the 3-day HW group which showed higher values of the SM muscle before packaging (*p* < 0.05; 0 day) than the 3-day TN group. Compared with the LTL, the SM muscle showed a greater response in retail colour following exposure to a short-duration HW (1 day). Colour results of the SM muscle’s a* from lambs exposed to a 1-day HW showed more changes compared with their TN counterparts, but this response was not seen in the LTL. This difference in response of the two muscles to acute stress (1-day HW) could be attributed to the higher percentage of type 1 muscle fibers and lower type 2B/X fibers in SM vs. LTL muscle [32,33,34]. Previous research has reported that muscle with a high percentage of type 1 and type 2A (soleus) fibers had a more rapid and broader response to HS as signified by the rapid induction of heat shock protein, compared to Type 2B or 2X muscle [35]. 

Across all the results from this experiment, it is evident that short duration heatwaves (1- to 5-day HW) applied in our experiment had no impact on postmortem muscle metabolites and pH decline and very limited impact on retail meat quality in lambs. There is no published data reporting the impact of short duration heat exposure of less than 1 week on the meat quality or metabolism of small ruminants. Previous research investigating the impact of HS on small ruminant meat quality has reported that HS leads to higher dark-cutting frequency and higher ultimate pH when the high ambient temperature exposure period is longer than 1 month [2,4]. For example, et al [5] reported that seasonal HS (35 °C, 47% RH; 6 months) significantly decreased the L*, a* and b* of psoas major and minor muscles of Omani Somali goats and Somali Merino sheep when compared with cool-season feeding (21 °C, 59% RH). Gregory [2] also confirmed that the meat quality of animals is compromised more in summer than in other seasons. Macías Cruz [36] reported that 1 month of summer feeding (28.4 °C, 55.2%) had no detrimental effects on meat colour of hair breed sheep compared with the winter (19.2 °C, 41.7% RH). This was in accordance with the results reported by Archana et al. [37] where 1 month of HS (28 and 40 °C and 29–58% RH) only increased the ultimate pH and decreased L* of the LTL muscle of Osmanabadi goats, but had no impact on a*, b* and WHC. We have previously shown that the muscle pH and meat quality in lamb was not affected after 2 weeks or 1 week controlled HS exposure (28–40 °C, 30–40% RH) [9,38]. Similar to the current study, we had observed that the lambs’ physiological and blood gas parameters were significantly affected by HS but not the meat quality and muscle pH [26]. These results also agree with previous work of Lowe, et al. [7], who reported that 12 h of HS did not change sheep meat quality.

Overall, results from this study are in accordance with the previous study in goats which reported that despite the noticeable influence of transport on blood parameters indicating stress in live animals, this status did not decisively alter meat quality parameters [39,40]. While few studies suggest that transportation under hot conditions (42 °C; 6 h) may negatively impact ruminant meat quality and pH [41,42], the impact of physiological perturbations caused by exposure to short durations of HS may not always manifest in the characteristics of the meat. Nevertheless, data from this study suggest that there is need to better manage lambs during a HW to reduce impact on their physiological functions, regardless of the impact on meat quality. In this experiment, lambs were able to cope with the HW by eliciting physiological heat loss mechanisms (increased respiration rate and heart rate, etc.) and were able to maintain muscle glycogen above the threshold levels for normal pH decline [21,43]. From the limited effect result of muscle pH (1-day HW) and meat colour (1- and 3-day HW groups of LTL and SM), acute HS has limited effects on muscle pH (HW1) and meat colour (HW3 and HW1 of LTL and SM respectively) as was seen here as the lambs may not have enough opportunity to adapt to acute stress. It may take 2–3 days for lambs to adapt to an ambient temperature change, even if the lamb’s physiological and blood parameters were changed [26]. The number of published studies focused on the relationship between short-term HS (less than 1 month) and meat quality is very limited and does not provide conclusive evidence. Furthermore, some of those studies include animals of different breeds with different heat resilience, and some studies have different exposure conditions of temperature and RH making it even more difficult to make comparisons (see review by Zhang, et al [3]). Therefore, a comprehensive study involving larger populations is required to demonstrate the impact of HS, of different durations and severity, on sheep meat quality.

## 4. Conclusions

Short duration HWs (1 to 5 days) did not affect the meat quality of lambs despite the significant perturbations in their normal physiology which is reflective of the animals’ adaptive response to HS. These changes in the physiological parameters were elicited by sheep to cope with HS but were not severe enough to influence muscle glycogen levels at slaughter, pH decline and meat quality. Therefore, exposure of lambs to short duration HWs over summer may not directly impact their meat quality provided lambs are not exposed to any other stressor such as nutritional stress or transportation stress.

## Figures and Tables

**Table 1:** The effect of temperature (thermoneutral, TN vs. heatwaves, HW) and heat exposure duration (1 vs. 3 vs. 5 days) on drip, purge and cooking loss of longissimus thoracis et lumborum (LTL) and semimembranosus (SM) muscle in 4 days’ overwrap retail display in lambs (*n* = 12 per group).

	1 Day		3 Days		5 Days		SED ^1^	*p*-Value		
	TN	HW	TN	HW	TN	HW		Temp. ^2^	Day	T × D ^3^
LTL										
Drip loss	2.02 ^a^	1.65 ^ab^	2.10 ^a^	1.74 ^ab^	1.95 ^a^	1.33 ^b^	0.287	0.01	0.36	0.77
Purge loss/%	2.47	2.34	2.54	2.44	2.32	2.45	0.194	0.24	0.17	0.97
Cooking loss 0 day/%	19.9	19.8	20.6	20.9	20.5	18.9	1.22	0.50	0.41	0.50
Cooking loss 4 days/%	22.8	23.8	23.6	23.2	22.7	23.3	0.97	0.46	0.73	0.62
SM										
Drip loss	1.55 ^a^	1.23 ^b^	1.42 ^a^	1.36 ^ab^	1.22 ^b^	1.07 ^b^	0.157	0.04	0.07	0.52
Purge loss/%	3.43 ^a^	2.62 ^b^	2.96 ^ab^	2.83 ^ab^	2.76 ^b^	2.36 ^b^	0.332	0.02	0.13	0.36
Cooking loss 0 day/%	18.8 ^ab^	18.9 ^ab^	20.1 ^a^	18.4 ^ab^	17.9 ^ab^	16.5 ^b^	1.41	0.24	0.11	0.63
Cooking loss 4 days/%	22.8	21.9	21.9	22.2	21.1	22.3	1.13	0.73	0.73	0.42

Values are mean ± SED; letters a and b in the same row mean a significant difference at 5% level of LSD; ^1^ SED = Standard error of the difference of means; ^2^ Temp. = Temperature; ^3^ T × D = Temperature × day.

**Table 2:** The effect temperature (thermoneutral, TN vs. heatwaves, HW) and heat exposure duration (1 vs. 3 vs. 5 days) on Warner–Bratzler shear force (WBSF) of longissimus thoracis et lumborum (LTL) and semimembranosus (SM) muscles during 4 days’ overwrap retail display (*n* = 12 per group).

	1 Day		3 Day		5 Day		SED ^1^	*p*-Value		
	TN	HW	TN	HW	TN	HW		Temp. ^2^	Day	T × D ^3^
LTL										
WBSF 0 day	27.9	31.3	35.0	32.1	30.6	27.2	36.6	0.13	0.16	0.99
WBSF 4 days	23.9	25.9	30.0	28.6	29.9	26.4	35.3	0.64	0.21	0.55
SM										
WBSF 0 day	40.9 ^a^	36.9 ^ab^	33.1 ^b^	39.6 ^a^	33.5 ^b^	36.9 ^ab^	29.1	0.32	0.19	0.04
WBSF 4 days	26.6	26.0	23.6	25.3	24.8	26.2	20.0	0.45	0.43	0.70

Values are mean ± SED; letters a and b in the same row mean a significant difference at 5% level of LSD; ^1^ SED = Standard error of the difference of means ^2^ Temp. = Temperature; ^3^ T × D = Temperature × day.

## Data Availability

The datasets generated for this study are available on request to the corresponding author.
